# Problem solving, loneliness, depression levels and associated factors in high school adolescents

**DOI:** 10.12669/pjms.325.10656

**Published:** 2016

**Authors:** Ummugulsum Sahin, Filiz Adana

**Affiliations:** 1 Ummugulsum Sahin, Uludag University Medical Faculty Hospital of Psychiatric Nurse, Bursa, Turkey; 2 Filiz Adana, Department of Public Health Nursing, School of Nursing, Adnan Menderes University, Aydin, Turkey

**Keywords:** Adolescent, Depression, High School Student, Loneliness, Problem Solving

## Abstract

**Objectives:**

To determine problem solving, loneliness, depression levels and associated factors in high school adolescents.

**Methods:**

This cross-sectional study was conducted in a city west of Turkey (Bursa) in a public high school and the population was 774 and the sampling was 394 students. Students to be included in the study were selected using the multiple sampling method. A personal Information Form with 23 questions, Problem Solving Inventory (PSI), Loneliness Scale (UCLA), Beck Depression Inventory (BDI) were used as data collection tools in the study. Basic statistical analyses, t-test, Kruskall Wallis-H, One Way Anova and Pearson Correlation test were used to evaluate the data. Necessary permissions were obtained from the relevant institution, students, parents and the ethical committee.

**Results:**

The study found significant differences between “problem solving level” and family type, health assessment, life quality and mothers’, fathers’ siblings’ closeness level; between “loneliness level” and gender, family income, health assessment, life quality and mothers’, fathers’, siblings’ closeness level; between “depression level” and life quality, family income, fathers’ closeness level.

**Conclusion:**

Unfavorable socio-economic and cultural conditions can have an effect on the problem solving, loneliness and depression levels of adolescents. Providing structured education to adolescents at risk under school mental health nursing practices is recommended.

## INTRODUCTION

Adolescence is a period where physical and emotional problems are experienced, which starts with sexual and psychosocial maturation and ends when the person has developed a sense of identity and become socially productive. This period is characterised with biological, psychological and social developmental changes.^1^,^2^ In order for an adolescent to grow up to be a healthy adult, he/she needs to have a healthy adolescence.^3^

In their daily lives, adolescents experience many problems caused by external and internal factors in their relations with their peers and adults, which they have to solve. Solving these problems successfully, eliminating encountered challenges and troubles has a positive influence in adolescents to adapt to life.^4^,^5^

One of the stages of life where a person feels the most lonely is adolescence. Studies report that socioeconomic levels, peer and family relations of adolescents have an effect on their level of loneliness.^6^

It is widely known that for an adolescent to adapt to the society he should be able to build healthy relations with his environment, be hopeful about the future and know how to deal with problems. The role of preventive services under the scope of nursing services based on scientific ground is major. “Provides protective intervention in the event of risky behaviour that can be seen in adolescence” is included in the job description of public health nurse pursuant to the Regulation on Nursing no 27515 dated 08.03.2011.^7^ Therefore identifying problem solving, loneliness and depression levels in adolescents is very important to decide on protective interventions.

This was a cross-sectional study which wanted to determine problem solving, loneliness, depression levels and associated factors in high school adolescents.

## METHODS

This was a cross-sectional study which was conducted during the academic year of 2013-2014 in a city west of Turkey. In the population of 774 students, it was planned to have 358 people with 99% confidence interval using sampling from finite population method and when the pattern effect was accepted as 1.2, the number of students reached to 394. Multiple sampling method was used for sampling.

Questionnaires were used in the classrooms and under supervision and lasted a total of 30 minutes. It is a self-report questionnaire and the privacy of the students was protected during the study. A personal Information Form, Problem Solving Inventory (PSI), Loneliness Scale (UCLA), Beck Depression Inventory (BDI) were used as data collection tools in the study.

### Personal Information Form

The Personal Information Form was developed based on the opinions of four experts and based on the findings in the literature.^3^,^6^

### Problem Solving Inventory (PSI)

The 6-point likert type inventory was adapted into Turkish by Işık Savaşır, Nail Şahin, Nesrin H. Şahin and Paul Heppner (1993). The inventory’s rating scale is between 32-192. Higher scores in the scale represent lower problem solving skills and lower scores represent higher problem solving skills. The Inventory consists of Problem Solving Confidence (PSI-C); Approach-Avoidance Style (PSI-AA) and Personal Control (PSI-PC).

### UCLA Loneliness Scale

The validity and reliability study of the Turkish version of the scale which was developed by Russel, Peplau and Ferguson (1878) to evaluate loneliness level of subjects was done by Demir (1989). The highest score in the scale is 80 and the lowest score is 20. The higher scores represent higher level of loneliness.

### Beck Depression Inventory (BDI)

The Inventory developed by Beck et al. in 1974 has a scoring scale of 0-40. The inventory consists of three sub-dimension namely emotional (BDI-E), motivational (BDI-M) and cognitive (BDI-C). The validity and reliability study of the Turkish version was done by Seber et al. (1993).

### Statistical Analysis

The data was evaluated with the SPSS statistics package program (Version 15, Chicago IL, USA) and using Windows XP operating system and basic statistical analyses, t-test, Kruskall Wallis-H, One Way Anova and Pearson Correlation test were used to evaluate the data. The statistical significance was accepted at the level of p <0.05.

### Ethical Considerations of the Research

Necessary permissions were obtained from the relevant institution, students, parents and the ethical committee. This study which was supported by Adnan Menderes University with 14010 project code was derived from post-graduate thesis.

## RESULTS

The mean age of the students included in the study was 16.41±1.21 and 84.1% were female and 15.6% were male. 84.5% of the students have nuclear families and 68.9% of the students’ families have balanced income-expenditure. About 69.5% of the student reported their father’s and 51.5% reported that their mother’s education level is middle school and higher and 71.4% of the students reported that they are close with their father, 90.1% reported that they are close with their mother. 93.42% of the students reported that their life quality is good and 53.8% reported that their health is good.

In the study PSI-C mean scores of the students who have nuclear families, PSI-C and PSI-AA mean scores of the students who have good health and PSI-C and PSI-AA mean scores of those who report to have good quality of life were found to be low.

PSI-C, PSI-PS and PSI-AA mean scores of the students who describe their fathers’ closeness level as good and PSI-AA mean scores of the students who describe their mothers’ closeness level as good closeness level were low.

**Table-I:** Comparison of Students’ Characteristics with the Mean Scores in PSI and Sub-Scales.

*Characteristics/PSI*			*PSI-Confidence (PSI-C)*		*PSI-Personal Control (PSI-PC)*		*PSI-Approach-Avoidance (PSI-AA)*	

		*n*	*M±SD*	*t,U p*	*M±SD*	*t,U p*	*M±SD*	*t,U p*
Family Type	Nuclear Family	333	31.63±8.95	2.728	21.20±4.06	1.63	51.40±9.88	1.058
	Extended Family	51	35.30±8.07	0.007	22.20±3.44	0.103	52.98±9.28	0.291
Health Assessment	Poor	181	29.47±8.37	5.82	21.06±4.23	1.31	49.80±10.02	3.44
	Good	213	34.53±8.80	0.001	21.58±3.70	0.18	53.14±9.29	0.001
Life Quality	Poor	26	35.88±7.11	3606.500	21.53±4.30	4722.000	54.80±10.08	3604.500
	Good	370	31.93±9.02	0.033	21.32±3.94	0.000	51.37±9.72	0.032
Fathers’ Closeness Level	Poor	114	35.18±9.58	4.262	22.08±3.69	2.258	54.49±8.74	3.742
	Good	280	31.02±8.37	0.001	21.08±4.05	0.024	50.47±9.91	0.001
Mothers’ Closeness Level	Poor	39	35.66±8.64	2.588	21.89±3.46	0.919	56.28±7.04	3.189
	Good	355	31.77±8.93	0.10	21.28±4.02	0.359	51.07±9.92	0.002

p<0.05

No significant relationship was found between the genders, income level of the family, father’s education, mother’s education and whether father and mother are both alive and together and the mean scores of the PSI and sub-scales.

Male students and those who reported to have less income compared to expenditure found to have higher UCLA mean scores. The UCLA mean score of the students with good health and with poor life quality. The students who reported that the closeness level of father and closeness level of mother were found to have higher UCLA mean scores. No significant relation was found between the family structure, father’s education, mother’s education and the mean scores of the UCLA.

BDI-T; BDI-E, BDI-M and BDI-C mean scores of the students who report that they have good quality of life, BDI-C mean scores of those who have higher income than their expenditures and BDI-E, BDI-M and BDI-C mean scores of those who report a close relation with their father were low.

No significant difference was found between the gender, family structure, father’s education, mother’s education, mother’s closeness level and the mean scores of the PDI and sub-scales.

## DISCUSSION

Several variables such as Personal characteristics, family structure, relations in the family, healthcare condition and quality life have an effect on the problem solving level.^4^,^5^,^8^ People with good family support learn to deal with the problems they encounter. Additionally the harmony in family relations make them feel safe.

**Table-II:** Comparison of Students’ Characteristics with UCLA Mean Score.

*Characteristics /UCLA*		*n*	*M±SD*	*t, ANOVA, U, p*	
Gender	Female	331	38.74±9.84	2.022	0.044
	Male	63	41.49±10.17		
Family Income	Low Income	63	42.53±9.51	5.38	0.001
	Equal Income	273	38.84±9.77		
	High Income	58	36.91±10.50		
Health Assessment	Poor	181	36.91±10.07	4.288	0.001
	Good	213	41.12±9.41		
Life Quality	Poor	26	47.03±9.29	2548.500	0.001
	Good	370	38.62±9.75		
Fathers’ Closeness Level	Poor	112	41.71±9.62	3,309	0.001
	Good	280	38.10±9.79		
Mothers’ Closeness Level	Poor	39	44.69±9.31	3.712	0.001
	Good	355	38.55±9.85		

p<0.05

**Table-III:** Comparison of Students’ Characteristics with the Mean Scores in BDI and Sub-Scales.

			*BDI-Emotional (BDIE)*		*BDI-Motivational (BDI-M)*		*BDI-Cognitive (BDI-C)*		*BDI-Total (BDI-T)*	

*Characteristics /BDI*		*n*	*M±SD*	*t, ANOVA, U, p*	*M±SD*	*t, ANOVA, U, p*	*M±SD*	*t, ANOVA, U, p*	*M±SD*	*t, ANOVA, U, p*
Family Income	Low Income	63	0.65±1.13	1.51 0.221	2.95±2.35	1.59 0.202	2.11±1.34	4.00 0.018	6.23±4.61	2.25 0.100
	Equal Income	273	0.80±1.29		2.49±2.12		1.71±1.32		5.52±4.38	
	High Income	58	0.51±0.99		2.29±2.04		1.43±1.40		4.55±4.08	
Life Quality	Poor	26	1.80±1.60	2552.00 0.001	4.30±2.05	2585.50 0.001	2.46±1.44	3330.50 0.007	9.42±4.96	2455.50 0.001
	Good	370	0.66±1.16		2.42±2.11		1.69±1.33		5.23±4.22	
Fathers’ Closeness Level	Poor	112	0.99±1.46	2.726 0.007	3.26±2.36	4.301 0.001	2.09±1.46	3.487 0.001	6.90±4.97	4.186 0.001
	Good	280	0.62±1.08		2.25±1.99		1.58±1.25		4.90±3.94	
Health Assessment	Poor	183	0.51±0.98		2.00±1.98		1.37±1.20		4.27±3.84	
	Good	213	0.93±1.37	3.449 0.001	3.02±2.19	4.806 0.001	2.06±1.39	5.234 0.001	6.57±4.56	5.370 0.001

p<0.05

In our study, PSI-Confidence mean scores of the students who have nuclear families are lower when compared to those who have large families and the problem solving skills of the students who are close with their mother and father are found to be high. In their study, Arslan and Kabasakal^8^ concluded that there is a significant difference between problem solving skills of the students and their parent’s attitudes. Parent’s attitude is a variable that has a major effect on a child’s behaviour. In that sense, it is possible to comment that problem solving skills of a child is formed in the family and with the help of the child’s parents.

In our study, students who reported to have good health had lower PSI-C and PSI-AA mean scores vs. students who reported poor health. Health problems can decrease a person’s ability to solve problems.

No significant relationship was found between the income level of the family, parent’s education and PSI and sub-scale. In the study of Gundogdu it was found that students’ level of perception of their problem solving skills did not show any significant difference based on the independent variables; mother’s education level and father’s education level.^9^ Our study findings are similar to those of Gundogdu’s^9^ study. While intense emotional conflicts in adolescence can cause loneliness in adolescents, the level of loneliness in this period of life can differ depending on personal characteristics.

In this study the loneliness level of male students was found to be higher than that of female students. In their study, Shevlin et al.^10^ concluded that women feel more lonely when compared to men. Civitci et al.^11^ did not find any significant difference in loneliness levels and genders in their study.

This study was conducted in a region in Turkey where women are raised to more dependent but men are raised to be more independent. When we consider these cultural aspects, it was an expected result based on the fact that social relations are more important for women and men have generally less intense relations with the people around them.

In our study, students who have lower income compared to their expenditure found to have higher loneliness levels. In their study, Shevlin et al.^10^ concluded that people whose economic condition was poor felt more lonely when compared o those who had better economic conditions. It can be concluded that the adolescent felt lonely due to the effect of lower income of the family on the adolescent’s socialization possibilities.

This study also showed that those who reported to have poor quality of life found to have higher loneliness level. When people have negative perceptions about their quality of life they can also feel more lonely and the difference in loneliness levels can be caused by interpersonal relationships which is one of the components of the quality of life.

Loneliness levels of the students who reported to have close relations with their fathers and mothers were found to be low. In their study Appel et al.^12^ found that loneliness had an effect on the parent-adolescent communication and perceived family support, Corsano et al.^13^ found that good relations with the family improved well-being in adolescents and students with good family relations had lower level of loneliness. Our findings show similarities with the comparable studies found in the literature.

No significant relationship was found between the family structure, father’s education, mother’s education in terms of loneliness level. Duyan et al.^14^ concluded that students who had university graduate parents had lower loneliness levels. The difference in study findings can be caused by different cultural and socio-economical conditions of the study locations.

### Depression Level

Personal and family characteristics of an individual, when combined with the effects of social environmental can affect his/her depression level.

We observed significant difference between the income level and BDI-C scores. The findings of our study are similar to the finding of the study of Ozmen et al.^15^ In line with these findings, income levels can be considered to have a direct impact on the potentials of the student and affect their level of hope. Better economic levels can contribute to reduce concerns about the future and this can make students feel more positive about their expectations about the future.

In the study, students who reported to have good quality of life had lower BDI and sub-scale scores. In their study Ozmen et al.^15^ found that students who had a poor perception of the quality of life had higher depression levels. Our study findings are similar to the findings of Ozmen et al.^15^ study. Adolescents with a hopeful perception of life can be said to be more hopeful about the future.

Those who reported to be close to their fathers had lower BDI and sub-scale scores. While Tumkaya et al.^16^ observed that parents’ attitude did not have an effect on the depression levels of high school students; Vidinlioglu^17^ and Tufekciyasar^18^ reported that students with negligent parents had higher levels of depression. The findings of our study are contradictory with the findings of the study of Tumkaya et al.^16^ while they are similar with the findings of the studies of Vidinlioglu^17^ and Tufekciyasar^18^ A reassuring attitude of parents can help adolescents to have a more hopeful attitude towards life.

In our study, those who reported to have good health had lower BDI and sub-scale scores. In their study Ozmen et al.^15^ found that students who were not happy with their health had higher depression levels. It is possible to conclude that a good perception of health can improve hopes of the person for the future.

No significant difference was found between BDI and sub-scale scores and gender, family structure, income level, parents’ education level, mother and siblings’ closeness level. Tumkaya et al.^16^ and Stoddard et al.^19^ concluded that depression did not differ according to gender; however, Tokuc et al.^20^ found that socio-economical status had an effect on depression, Tumkaya et al.^16^ concluded that the number of family members was an effective variable in depression.

Tumkaya et al.^16^ did not find any significant difference between adolescents’ parents’ education levels and their depression levels. In their studies Ozmen et al.^15^ and Kolarcik et al.^21^ demonstrated that parents’ low level of education had a negative impact on the depression level.

### Limitation of the study

This study was designed to be a single-center study with a limited student sample.

## CONCLUSION

We found that there is positive correlation with depression and loneliness and the higher depression and loneliness levels are associated with lower problem solving levels. Unfavorable socio-economic and cultural conditions can have an effect on the problem solving, loneliness and depression levels of adolescents. Providing structured education to adolescents at risk under school mental health nursing practices are recommended.
